# Antioxidants selenomethionine and D-pantethine decrease the negative side effects of doxorubicin in NL/Ly lymphoma-bearing mice

**DOI:** 10.3325/cmj.2016.57.180

**Published:** 2016-04

**Authors:** Rostyslav R. Panchuk, Nadia R. Skorokhyd, Yuliya S. Kozak, Liliya V. Lehka, Vira V. Chumak, Sofya N. Omelyanchik, Valery A. Gurinovich, Andrey G. Moiseenok, Rostyslav S. Stoika

**Affiliations:** 1 Department of Regulation of Cell Proliferation and Apoptosis, Institute of Cell Biology NAS of Ukraine, Lviv, Ukraine; 2 Department of Biochemistry, Biological Faculty, Ivan Franko Lviv National University, Lviv, Ukraine; 3 Laboratory of Metabolomics, Institute of Biochemistry of Biologically Active Compounds, Grodno, Belarus

## Abstract

**Aim:**

To investigate the potential tissue-protective effects of antioxidants selenomethionine and D-pantethine applied together with doxorubicin (Dx) on NK/Ly lymphoma-bearing mice. The impact of this chemotherapy scheme on animal survival, blood cell profile, hepatotoxicity, glutathione level, and activity of glutathione-converting enzymes in the liver was compared with the action of Dx applied alone.

**Methods:**

The hematological profile of animals was studied by the analysis of blood smears under light microscopy. Hepatotoxicity of studied drugs was evaluated measuring the activity of alanine aminotransferase (ALT) and aspartate aminotransferase (AST) enzymes, De Ritis ratio, and coenzyme A fractions by McDougal assay. Glutathione level in animal tissues was measured with Ellman reagent, and the activity of glutathione reductase, transferase, and peroxidase was measured using standard biochemical assays.

**Results:**

D-pantethine (500 mg/kg) and, to a lower extent, selenomethionine (600 µg/kg) partially reduced the negative side effects (leukocytopenia and erythropenia) of Dx (5 mg/kg) in NK/Ly lymphoma bearing animals on the 14th day of their treatment. This increased animal survival time from 47-48 to 60+ days and improved the quality of their life. This ability of D-pantethine and selenomethionine was realized via hepatoprotective and immunomodulating activities. D-pantethine also restored the levels of acid-soluble and free CoA in the liver of tumor-bearing animals, while selenomethionine caused the recovery of glutathione peroxidase levels in the liver, which was significantly diminished under Dx treatment. Both compounds decreased glutathione level in the liver, which was considerably induced by Dx.

**Conclusions:**

Antioxidants selenomethionine and D-pantethine partially reversed the negative side effects of Dx in NK/Ly lymphoma-bearing mice and significantly increased the therapeutic efficiency of this drug in tumor treatment.

The vast majority of all agents used to directly kill cancer cells (ionizing radiation, most chemotherapeutic agents, and some targeted therapies) work through either directly or indirectly generating reactive oxygen species that block the key steps in the cell cycle (1). That is why in the last two decades the use of dietary supplements with antioxidant activity in cancer therapy has become extremely popular with an aim to decrease potential side effects of cancer chemotherapy on the organism of cancer patients. It is believed that the application of antioxidants can decrease side effects of drugs on healthy tissues of patients, but have a little influence on the therapeutic toxicity of these drugs toward tumors.

Despite nearly two decades of investigations of use of dietary antioxidant supplementation during conventional chemotherapy and radiation therapy, controversy remains about the efficacy and safety of this complementary treatment. Different sources estimate the actual usage of antioxidant supplements by cancer patients in the range between 13% and 87% (2-4). However, the use of antioxidant supplements by patients undergoing chemotherapy has been criticized due to the concerns that antioxidants may interfere with the mechanism of action of chemotherapy and subsequently decrease its efficacy, protecting both healthy and tumor cells from oxidative damage (5,6). However, other data suggest that antioxidants can protect normal tissues from chemotherapy- or radiation-induced damage without decreasing tumor control, as well as alleviate toxic side effects of drugs, allowing patients to tolerate chemotherapy for the full course of treatment and possibly at higher doses (7). As a result, patients may have better tumor response rates and increased chances of survival.

The most popular antioxidant supplements used in clinics include vitamins A,C,E, melatonin, selenium derivatives, N-acetylcysteine, and glutathione (8). Inside the cells these compounds act either as reducers or lipid chains breakers (melatonin, NAC, Vitamin E, GSH, beta carotene, and vitamin C) or essential elements of antioxidant enzymes formed by combining with a protein to form selenoproteins (selenium, GSH) (8). In some cases antioxidants may act as metal chelators (Vitamin C, ellagic acid, and dexrazoxane) or cellular protectors from free radical attack (vitamins A, C, E. and melatonin) (8).

Anthracycline doxorubicin (Dx) is one of the most widely used chemotherapeutic agents with a broad spectrum of activity. Dx is commonly applied for the treatment of a large number of tumor types, including non-Hodgkin’s and Hodgkin’s lymphoma, multiple myeloma, as well as lung, ovarian, gastric, thyroid, breast, sarcoma, and pediatric cancers (9). However, Dx use in cancer treatment is limited by serious shortcomings, including cumulative dose-dependent cardiotoxicity, myelosuppression, and hepatotoxicity (10,11). It is considered that these negative side effects are caused by excessive production of reactive oxygen species (ROS) in the mitochondria of targeted cells. Thus, lowering ROS production under Dx treatment by specific antioxidants should decrease these side effects, allowing an increase in the maximum therapeutic dose of the drug, improvement of quality of life of cancer patients, and their survival rates.

One of the ways to solve this problem is the use of antioxidant dietary supplements, which can counteract free radicals and prevent their action causing tissue and organ damage (12). The main mechanisms of antioxidants action are the suppression of free radicals formation, inhibition of chain initiation and/or propagation, reparation and reconstitution of cell membranes, or key antioxidant enzymes’ functioning as coenzymes, thus greatly enhancing their activity (13).

We have previously reported (14) on a significant modulatory effect of selenium derivatives (sodium selenite and selenomethionine) and vitamin B_5_ precursor – D-pantethine – on cytotoxic activity of anticancer drug doxorubicin (Dx) *in vitro* toward various tumor cell lines. It was also demonstrated that selenomethionine (SeMet) and D-pantethine (D-Pt) partially decreased the side effects of Dx *in vivo* in healthy Wistar rats, however there are no data about their tissue-protective activity in animal tumor models. It is known that selenium plays an important role in the regulation of glutathione system, being a coenzyme of glutathione peroxidase (15), while D-Pt is a precursor of coenzyme A, involved in the majority of metabolic processes in the cell (16,17).

The main goal of current study was to investigate the therapeutic effect of joint application of SeMet and/or D-Pt with Dx *in vivo* in NK/Ly lymphoma. The impact of this chemotherapy scheme on animal survival, blood profile, hepatotoxicity, glutathione level, and activity of glutathione-converting enzymes in animal tissues was studied and compared with the action of Dx alone.

## Materials and methods

Studies of the biological activity of selenomethionine (SeMet), D-pantethine (D-Pt), and doxorubicin were conducted in 2015 at the animal facility of Institute of Cell Biology NAS of Ukraine (Lviv, Ukraine). 60 adult male Balb/c mice with 25-28 g weight were kept under standard vivarium conditions with constant access to the full feed and drinking water. Animals were divided into 10 groups with 6 mice in each.

Murine NK/Ly lymphoma was obtained from the tumor strain collection at R.E. Kavetsky Institute of Experimental Pathology, Oncology and Radiobiology, NAS of Ukraine, Kyiv. The ascites tumor was supported by transferring 0.2-0.3 mL of ascitic fluid (20-30 mln cells) from donor mouse into the abdominal cavity of the recipient mouse. Ascites from the tumor-bearing mice was obtained and transplanted on the 7-8th day after the inoculation. Tumor growth was controlled by everyday weighting of mice. The viability and number of cells in the ascitic fluid were checked by cell counting in the hemocytometric chamber in the presence of 0.05% trypan blue. The lymphoma cell vitality in the ascites used for transplantation was not lower than 98%.

**Figure 1:**
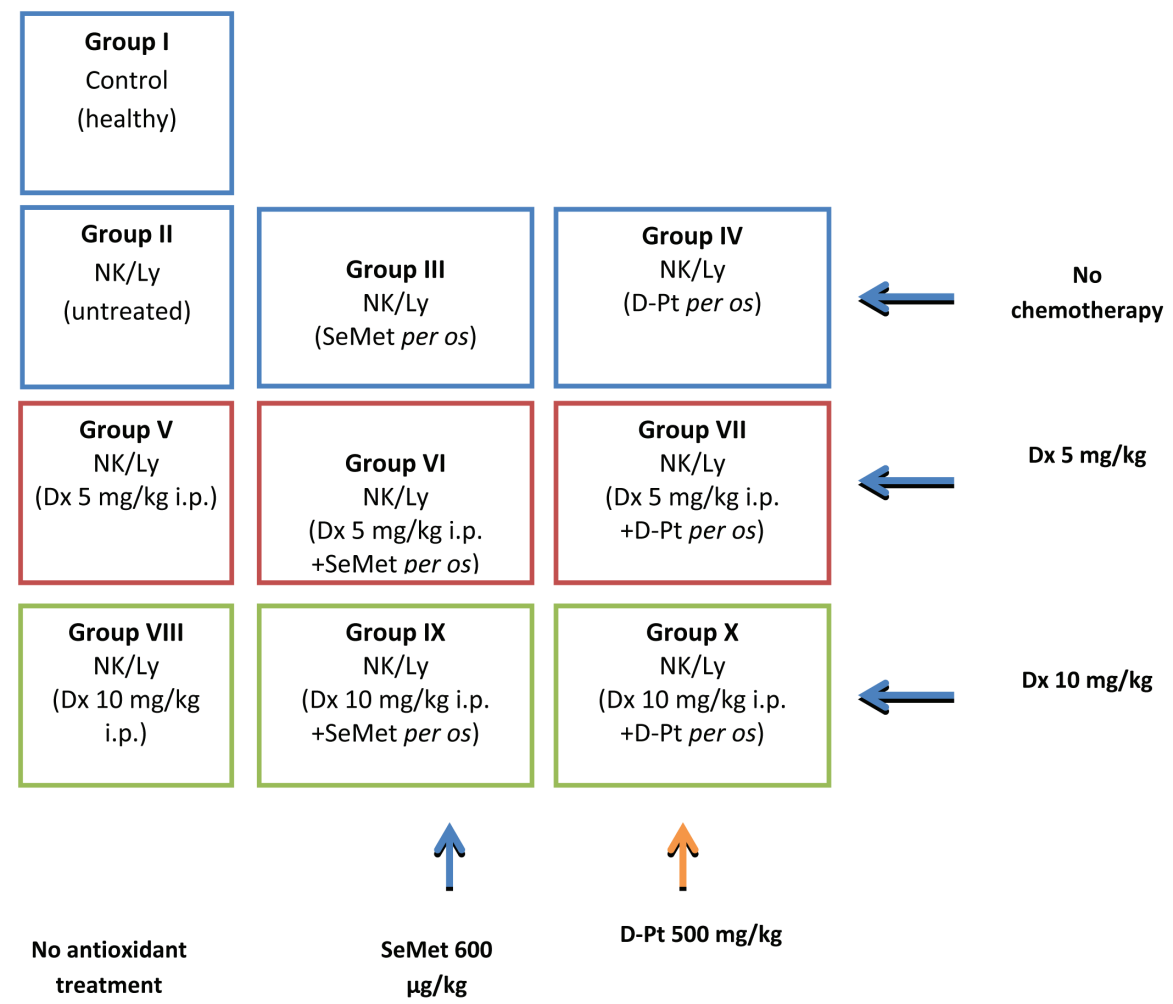
Mice treatment scheme by anticancer drug and antioxidants.

Organic selenium derivative Seleno-L-methionine (≥98% (TLC)) and D-pantethine (vitamin B_5_ precursor) were purchased from Sigma (St. Louis, MO, USA), and doxorubicin hydrochloride was obtained from Pfizer (New York, NY, USA). Antioxidants were dissolved in sterile 0.9% sodium chloride solution prior to *per os* treatment of animals. 1000 μg dose of L-selenomethionine/kg bw/d (equivalent to 400 μg selenium/kg bw/d) was reported to be a no-observed-adverse-effect-level (NOAEL) in a 13-week study in rats (18). At the same time, no genotoxicity and no carcinogenicity has been observed for D-Pt action, and no developmental toxicity in mice and rats at up to 600 mg/kg bw/d doses (19). In our study, animals received cumulative dose of 600 µg/kg of SeMet *per os* and 500 mg/kg of D-Pt *per os* from the 2nd till 20th day after tumor inoculation, which is 10-fold lower than NOEAL for both antioxidants, to exclude any potential side effects. Mice were divided into 10 experimental groups (Figure 1).

Animals were administered selenomethionine (60 µg/kg, cumulative dose 600 µg/kg) (groups 3, 6, 9) or D-Pt (50 mg/kg, cumulative dose 500 mg/kg) (groups 4,7,10) *per os* every second day, starting from 2nd till 20th day of tumor inoculation. The chemotherapy scheme was developed based on NCI recommendations (20) and other studies describing combined use of anticancer drugs and antioxidants (21,22). Mice from the first (control) and second (NK/Ly lymphoma, untreated) groups received simultaneously the equivalent volume of 0.9% sodium chloride solution in a similar mode. Doxorubicin in low (0.5 mg/kg, cumulative dose 5 mg/kg) and higher dose (1 mg/kg, cumulative dose 10 mg/kg) was injected intraperitoneally (i.p.) every second day starting from 2nd till 20th day of tumor inoculation to the animals from 5-7th and 8-10th groups, respectively. Treatment of animals with antioxidants in groups 6-7, 9-10 took place 1h before Dx injection. Tumor growth was monitored by daily weighting of animals, as increase in animal weight strongly correlates with NK/Ly tumor growth (23)

For studies of glutathione, CoA, and enzyme activities in tissues of experimental animals, additional group of animals was used in another experiment. 30 adult male Balb/c mice with 25-28 g weight were divided into 5 groups of 6 mice each: control (healthy), NK/Ly lymphoma (untreated), Dx 5 mg/kg, Dx 5 mg/kg+SeMet 600 µg/kg, and Dx 5 mg/kg+D-Pt 500 mg/kg. Drugs were administered in the same way as mentioned before. Animals were sacrificed at the 21st day after tumor inoculation by cervical dislocation, their organs were removed, washed in saline, and homogenized for further studies.

All *in vivo* experiments were conducted in accordance with the international principles of the European Convention for protection of vertebrate animals under the control of the Bio-Ethics Committee of the above mentioned institution (Protocol Nş 9/2015 from 1.09.2015 of the BioEthics committee of the Institute of Cell Biology, NAS of Ukraine).

For blood sampling, a small part of mouse tail was amputated, ~ 50 µL of blood was pumped into a test tube, and the wound was immediately disinfected with 70% alcohol. For red blood cell count 5 µL of blood were dissolved in 5 mL of isotonic NaCI solution (1:1000 dilution), while for leukocyte count 5 µL of blood was dissolved in 95 µL of 3% acetic acid solution (1:20 dilution). Erythrocytes were counted in 5 big squares (divided into 16 small each) of hemocytometric Goryaev’s chamber, while leukocytes were counted in 100 large squares, grouped by 4, under the Evolution 300 Trino microscope (Delta Optical, Mińsk Mazowiecki, Poland). The number of erythrocytes and leukocytes was calculated by standard formulas (24).

For blood smear preparation, 3 µL of blood was put at the edge of a slide, and then spread for 1.5 cm using another narrow polished slide, placed at a 45° angle. The obtained smears were dried at room temperature, then fixed with absolute methanol, and later rehydrated by subsequent washing in ethanol solutions with decreasing concentrations (96%, 75%, 50%, 25%, 12.5%). Finally, the smears were washed with distilled water, stained with Giemsa dye using standard protocol, and air-dried, after which they were ready for leukogram analysis.

Light microscopy counting of leukocytes performed under Evolution 300 Trino microscope (Delta Optical) on 90 × oil immersion objective. Cell counting was always done using the same system – half of the cells were counted in the upper half part of the smear, and the other 50% of cells were counted on the lower part of the smear. The percentage of certain types of white blood cells in each smear was determined after counting of at least 300 cells. The obtained values (due to differences in absolute numbers of cells in each counted smear) were normalized to 100%, and percent values of each leukocyte fraction were calculated as described in the work of Wilkinson et al (25).

For determination of aspartate aminotransferase activity, 10 µl of serum was mixed with 100 µL of substrate solution (2 mM α-ketoglutaric acid; 0.2 M D,L-aspartate in 0.1 M phosphate buffer pH 7.4), while in control tube 10 µL of distilled water were added instead of serum. The tubes were placed for 60 min at 37°C, and then 100 µL of 1mM solution of 2,4-dinitrophenylhydrazine was added to the samples and left for 20°C at room temperature. After this, 1 mL of 0.4 M sodium hydroxide solution was added to each sample for extra 10 min, and the optical density of the samples was measured using Helios Gamma spectrophomometer (Thermo Fisher Scientific, Waltham, MA, USA) at 540 nm wavelength. For alanine aminotransferase activity, the procedure was identical, except for substrate solution (2 mM α-ketoglutaric acid; 0.2 mM DL-alanine in phosphate buffer pH 7.4).

For better understanding of the role of studied antioxidants on liver functions in NK/Ly lymphoma bearing animals under Dx treatment, glutathione and coenzyme A levels, together with the activity of glutathione*-*related enzymes in the liver. were studied in more detail.

The measurement of glutathione reductase and glutathione transferase activity was performed according to Carlberg et al and Rice-Evans et al, respectively (26,27). Determination of glutathione and its redox potential in the liver was conducted as previously described (28). The analysis of free SH-groups in proteins from blood plasma was done as previously described (29).

Glutathione peroxidase activity was measured by two different assays based on use of various peroxide-containing substrates. In the assay described by Moin et al (30), t-BOOH was used as a substrate, while the activity of selenium-specific GPx was measured as described by Kruglikova et al (31), with hydrogen peroxide as the main substrate. For inhibition of catalase activity, sodium aside was added to the incubation mixture.

Identification of coenzyme A fractions was conducted as previously described (32,33). Study of the content of free form of coenzyme A (CoA-SH) and short chain acyl-CoA derivative (acetyl-CoA) in the rat liver was carried out using high-performance liquid chromatography HPLC assay on a HPLC instrument Agilent 1100/1200 (Agilent Technologies, Santa Clara, CA, USA). Homogenization of liver tissue samples was performed at 4°C using 4% HClO_4_ at a ratio of 1:6. The homogenates were centrifuged at 16 000 g for 15 min at 4°C and the obtained chlorine supernatants were adjusted to pH 5 with 20% NaOH. Prior to introduction into the chromatograph, the supernatant was filtered through a RC filter (0.45 µm, 13 mm) (Agilent Technologies). Zorbax SB-C_18_ chromatographic column, 150 × 3 mm, particle size 3.5 µm (Agilent Technologies) was used for HPLC.

All experiments were performed in triplicate and repeated 3 times. Statistical analysis of data due to low number of samples per group (n = 6) was conducted in GraphPad Prism software (GraphPad Software Inc. La Jolla, CA, USA) using non-parametric tests (2-way ANOVA with Bonferroni post-hoc tests, which compares replicate values by rows). The level of significance was set to be lower than 0.05.

## Results

### Selenomethionine and D-pantethine increase survival time of NK/Ly lymphoma bearing animals treated with Dx

**Figure 2:**
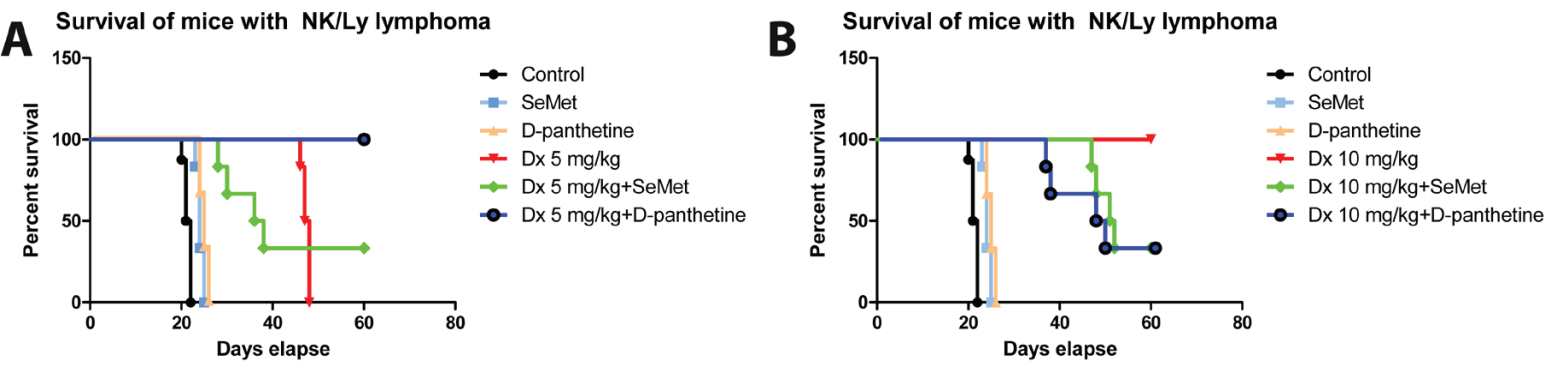
Survival of animals with NK/Ly lymphoma treated with various concentrations of Dx and antioxidant compounds.

**Figure 3:**
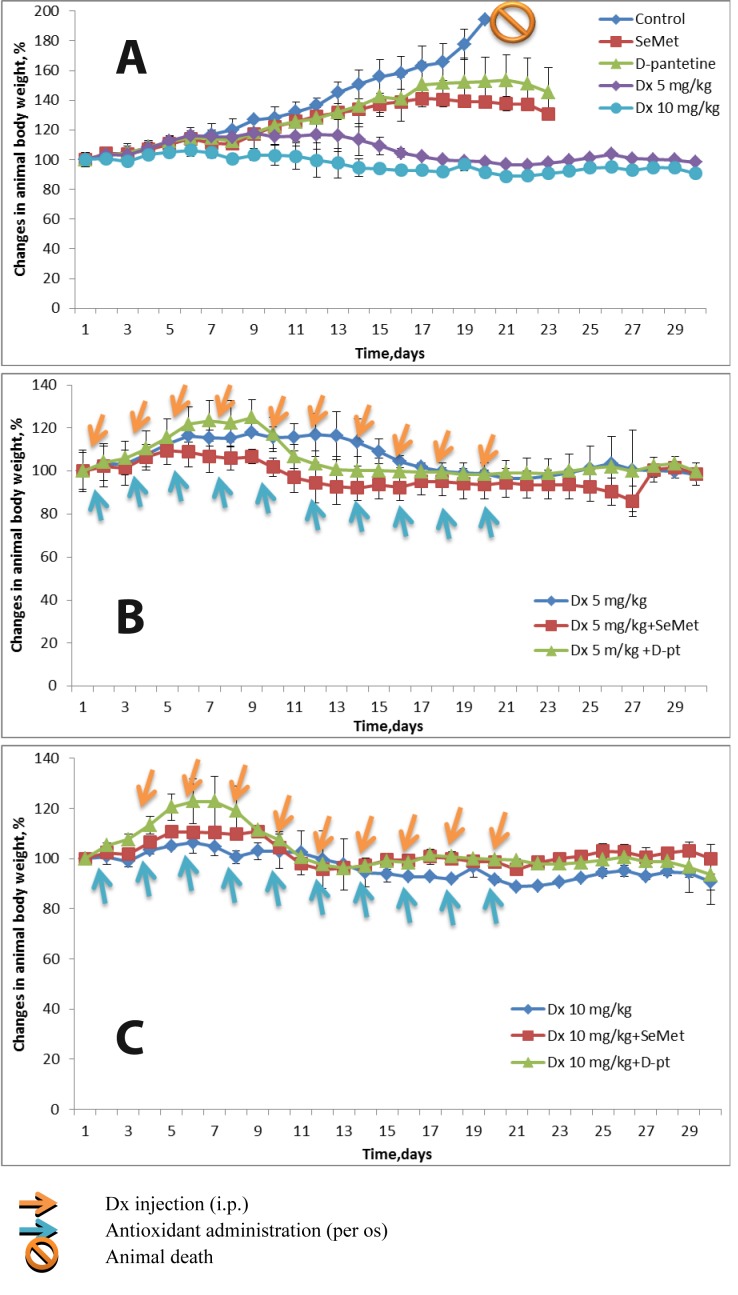
Changes in the weight of animals with NK/Ly lymphoma treated with various concentrations of Dx and antioxidant compounds.

Data on survival of animals treated with various cumulative doses of Dx (5 mg/kg and 10 mg/kg), SeMet (600 µg/kg), and/or D-Pt (500 mg/kg) are presented in Figure 2. Application of SeMet (cumulative dose 600 µg/kg) and D-Pt (cumulative dose 500 mg/kg) had a little impact on animal survival (up to 23-25 days). However, both compounds revealed moderate anticancer activity, as they decreased 2-fold the volume of ascitic fluid in comparison with the control group (*P* < 0.01 for SeMet at 19th day of tumor inoculation, but non-significant for D-Pt) (Figure 3A). Treatment of animals with Dx (5 mg/kg) significantly increased animal survival in comparison with the control – up to 48 days after tumor inoculation, but none of them survived till the end of experiment (60 days). Joint application of SeMet (600 µg/kg) and Dx (5 mg/kg) led to a complete remission in 33% of animals (survival >60 days) (Figure 2A). Combination therapy with D-Pt (500 mg/kg) and Dx (5 mg/kg) was even more effective, leading to tumor remission in 100% animals (Figure 2A), thus suggesting a prominent therapeutic efficiency of D-Pt in combination with low doses of Dx. These data were also supported by animal weight studies, which have shown an enhancement of anticancer activity of Dx by SeMet (*P* < 0.01 at 13th day) and D-Pt (*P* < 0.05 at 13th day) in the period from the 10th to 18th day after tumor inoculation (Figure 3B).

Higher doses of Dx (10 mg/kg), in contrast to lower doses (5 mg/kg), cured 100% animals with NK/Ly lymphoma (survival >60 days) (Figure 2B). However, in this case therapeutic effect of SeMet and D-Pt was much lower (*P* < 0.001) compared to their action in combination with 5 mg/kg dose of Dx, and only 33% animals treated with these antioxidants and Dx survived for more than 60 days, while other 67% died at the 36th-52nd day after tumor inoculation (Figure 2B). However, animal survival data contradict to animal weight studies (Figure 3C), which clearly show beneficial effects of dietary supplements, which neutralized the weight loss (5%-10%) observed in all Dx-treated animals.

### SeMet and D-pantethine decrease myelosuppressive effects of Dx in tumor-bearing animals

**Figure 4:**
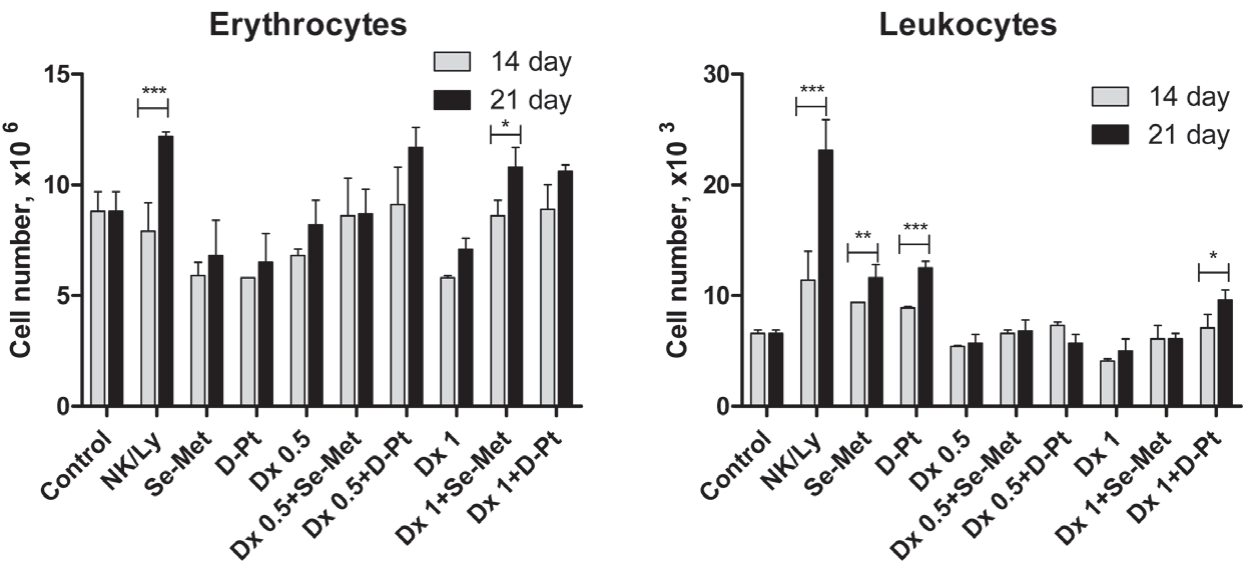
Comparison of the number of erythrocytes and leukocytes in NK/Ly lymphoma-bearing animals treated with various concentrations of Dx and antioxidant compounds, on the 14th and 21st day after tumor inoculation. *P < 0.05 related to 14th day, **P < 0.01 related to 14th day, ***P < 0.001 related to the 14th day.

**Figure 5:**
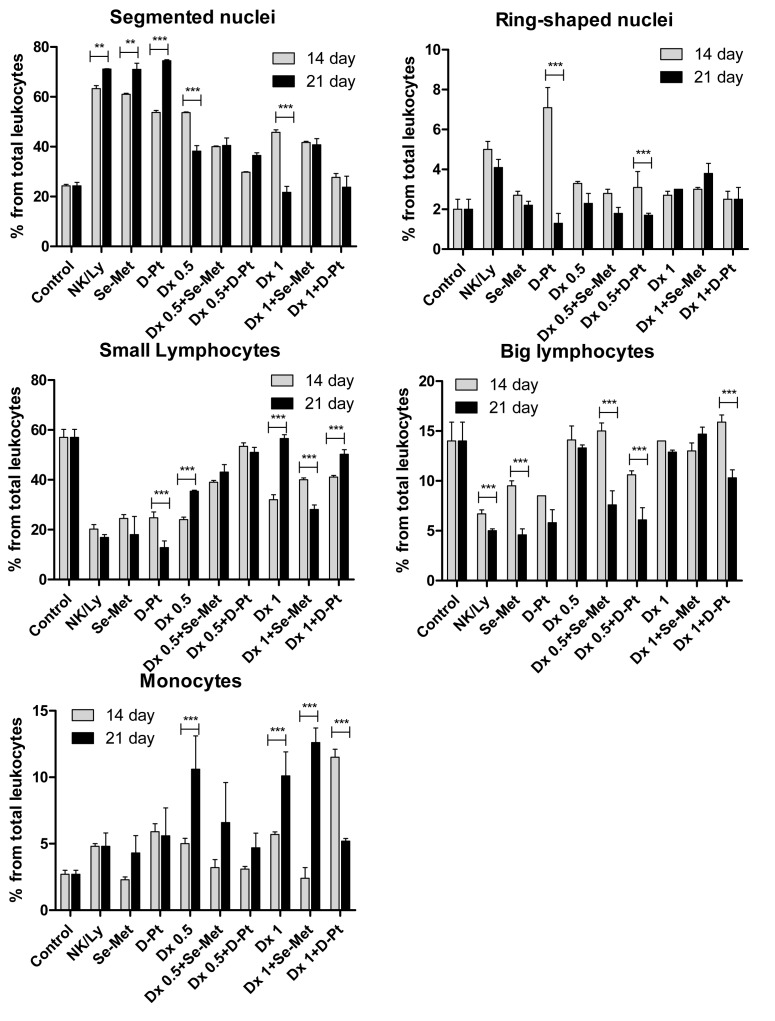
Changes in leukogram in NK/Ly lymphoma-bearing animals treated with various concentrations of Dx and antioxidant compounds, on the 14th and 21st day after tumor inoculation. **P < 0.01 related to the 14th day, ***P < 0.001 related to the 14th day.

For in-depth studies of the impact of antioxidants on myelosupressive activity of Dx blood smears were prepared from NK/Ly lymphoma-bearing animals at the 14th and 21nd day after tumor inoculation, and were compared with such smears, prepared from blood of healthy (control) animals (Table 1, Figure 4, Figure 5).

**Table 1:** Impact of doxorubicin, selenomethionine, and D-pantethine on blood formula of NK/Ly lymphoma bearing mice on the 14th day and 21st day after tumor inoculation (M±SD)

14th day							
Groups	WBC, (*10^3^/mL)	RBC, (*10^9^/mL)	SN (%)	RSN (%)	SL (%)	BL (%)	M (%)
Control (healthy)	6.6 ± 0.3	8.8 ± 0.9	24.3 ± 1.4	2.0 ± 0.5	57.0 ± 3.2	14.0 ± 1.9	2.7 ± 0.3
Group I (C, NK/Ly)	11.4 ± 2.6***	7.9 ± 1.3	63.3 ± 2.9***	5.0 ± 0.4	20.2 ± 1.8***	6.7 ± 0.4^***^	4.8 ± 0.2^**^
Group II (Se-Met)	9.4 ± 0.1**	5.9 ± 0.6***	61.0 ± 1.0^***^	2.7 ± 0.2	24.5 ± 1.5***	9.5 ± 0.5	2.3 ± 0.2
Group III (D-Pt)	8.9 ± 0.1**	5.8 ± 0.1***	53.8 ± 1.8^▲▲▲^	7.1 ± 1.0	24.8 ± 2.3***	8.5 ± 0.0	5.9 ± 0.6
Group IV (Dx 5)	5.4 ± 0.1^**^	6.8 ± 0.3	53.7 ± 0.6^▲▲▲^	3.3 ± 0.1	24.0 ± 1.0***	14.1 ± 1.4	5.0 ± 0.4
Group V (Dx 5+Se-Met)	6.6 ± 0.3^●^	8.6 ± 1.7	40.0 ± 0.8^●●●^	2.8 ± 0.2	39.0 ± 0.8^●●●^	15.0 ± 0.8	3.2 ± 0.6
Group VI (Dx 5+D-Pt)	7.3 ± 0.3^●●●^	9.1 ± 1.7^●^	29.8 ± 0.4^●●●^	3.1 ± 0.8	53.4 ± 1.4^●●●^	10.6 ± 0.4	3.1 ± 0.2
Group VII (Dx 10)	4.1 ± 0.2^***^	5.8 ± 0.1***	45.8 ± 2.3^▲▲▲^	2.7 ± 0.2	32.0 ± 2.0^▲▲▲^	14.0 ± 0.0	5.7 ± 0.2
Group VIII (Dx 10+Se-Met)	6.1 ± 1.2^##^	8.6 ± 0.7^###^	41.7 ± 1.2	3.0 ± 0.1	40.0 ± 0.8^###^	13.0 ± 0.8	2.4 ± 0.8^###^
Group IX (Dx 10+D-Pt)	7.1 ± 1.2^###^	8.9 ± 1.1^###^	27.7 ± 3.8^###^	2.5 ± 0.4	41.1 ± 0.7^###^	15.9 ± 0.7	11.5 ± 0.6^###^
							
21st day							
Groups	WBC, (*10^3^/ml)	RBC, (*10^9^/ml)	SN (%)	RSN (%)	SL (%)	BL (%)	M(%)
Control (healthy)	6.6 ± 0.3	8.8 ± 0.9	24.3 ± 1.4	2.0 ± 0.5	57.0 ± 3.2	14.0 ± 1.9	2.7 ± 0.3
Group I (C, NK/Ly)	23.1 ± 2.8***	12.2 ± 0.2	71.2 ± 0.1***	4.1 ± 0.4	16.9 ± 1.1***	5.0 ± 0.2^***^	4.8 ± 1.0^**^
Group II (Se-Met)	11.6 ± 1.2***	6.8 ± 1.6	71.0 ± 2.5	2.2 ± 0.2	18.0 ± 7.3***	4.6 ± 0.6	4.3 ± 1.3
Group III (D-Pt)	12.5 ± 0.6***	6.5 ± 1.3	74.5 ± 0.4	1.3 ± 0.5	12.8 ± 2.7***	5.8 ± 1.3	5.6 ± 2.1
Group IV (Dx 5)	5.7 ± 0.8	8.2 ± 1.1	38.2 ± 2.2^▲▲▲^	2.3 ± 0.5	35.4 ± 0.4^▲▲▲^	13.3 ± 0.3	10.6 ± 2.5^▲▲▲^
Group V (Dx 5+Se-Met)	6.8 ± 1.0	8.7 ± 1.1	40.5 ± 3.0	1.8 ± 0.3	43.1 ± 3.0^●●●^	7.6 ± 1.4	6.6 ± 3.0
Group VI (Dx 5+D-Pt)	5.7 ± 0.8	11.7 ± 0.9^●●●^	36.5 ± 1.1	1.7 ± 0.1	51.0 ± 2.0^●●●^	6.1 ± 1.2	4.7 ± 1.1^●●●^
Group VII (Dx 10)	5.0 ± 1.1	7.1 ± 0.5	21.7 ± 2.4^▲▲▲^	3.0 ± 0.0	56.6 ± 1.5^▲▲▲^	12.9 ± 0.2	10.1 ± 1.8^▲▲▲^
Group VIII (Dx 10+Se-Met)	6.1 ± 0.5	10.8 ± 0.9^###^	40.8 ± 2.4^###^	3.8 ± 0.5	28.1 ± 1.8^###^	14.7 ± 0.7	12.6 ± 1.1
Group IX (Dx 10+D-Pt)	9.6 ± 0.9^###^	10.6 ± 0.3^###^	23.8 ± 4.4	2.5 ± 0.6	50.2 ± 1.9^##^	10.3 ± 0.8	5.2 ± 0.2^###^

RBC – red blood cells; WBC – while blood cells; RSN – neutrophils with ring-shaped nuclei.

SN – neutrophils with segmented nuclei; SL – small lymphocytes; BL – big lymphocytes; M – monocytes.

* *P* < 0.05 related to control, **P < 0.01 related to control, ***P < 0.001 related to control, ^▲▲▲^ *P* < 0.001 related to control(NK/Ly); ^●^*P* < 0.05 related to Dx 5 mg/kg, ^●●●^*P* < 0.001 related to Dx 5 mg/kg, ^##^– *P* < 0.01 related to Dx 10 mg/kg; ^###^– *P* < 0.001 related to Dx 10 mg/kg.

Despite the therapeutic effect of Dx on NK/Ly lymphoma, this drug in 5 mg/kg dose also caused severe side effects on hematopoietic system, namely inducing erythropenia. Both SeMet and D-Pt restored the number of erythrocytes almost to the number of control samples (Table 1, Figure 4). The same tendency was observed for changes in leukocytes number, which also significantly decreased under Dx treatment (*P* < 0.001 at 14th day), while both SeMet and D-Pt restored these indices almost to the control level on the 14th and 21st day (Table 1, Figure 4).

The development of NK/Ly lymphoma significantly (*P* < 0.001) increased the level of segmented neutrophils, while Dx (5 mg/kg) decreased this index both at the 14th and 21st day after tumor inoculation, suggesting a therapeutic effect of this drug (Table 1, Figure 5). However, combined therapy with SeMet (600 µg/kg) and Dx (5 mg/kg) led to 20% more decrease in the level of segmented neutrophils, while D-Pt (500 mg/kg) decreased this index even stronger (1.8-fold lower compared to the action of Dx alone).

The number of small lymphocytes (T- and B-lymphocytes) is another important index that is rapidly decreased in tumor-bearing animals compared to control (Table 1, Figure 5). Dx (5 mg/kg) partially restored this index at the 14th and 21st day after tumor inoculation, while SeMet and D-Pt applied together with this drug, increased it 1.5- and 2.2-fold on the 14th day, respectively (Table 1). On the 21st day, such an effect of antioxidants was also preserved (reversal of the number of small lymphocytes almost to the control level), but due to further increase of this index in Dx group, the differences between them were less expressed (Table 1, Figure 5).

The same tendencies were observed when animals were treated with antioxidants and higher dose of Dx (10 mg/kg) (Table 1, Figure 4, Figure 5). Dx (10 mg/kg) caused even more severe side effects when it comes to the number of erythrocytes compared to control, while SeMet effectively restored this index to the control level, and D-Pt demonstrated even higher efficiency (Table 1). Another specific feature of Dx is development of monocytosis in treated animals, as observed using both drug doses on the 21st day after tumor inoculation, and D-Pt was the only compound that effectively normalized this index to the control level (Table 1, Figure 5).

We did not found any major differences in the impact of Dx and Dx + antioxidants on the ratio of segmented neutrophils and small lymphocytes, due to fact that Dx (10 mg/kg) itself restored these indices almost to the control level, compared to the untreated animals (Table 1). Thus, both antioxidants stopped leukopenia, erythrocytopenia, and monocytosis induced by Dx, but they had no immunomodulating impact (due to high comparative basis of Dx alone) on the ratio of segmented neutrophils and small lymphocytes under action of high doses of this drug.

### SeMet and D-pantethine partially modulate hepatotoxicity of Dx

**Figure 6:**
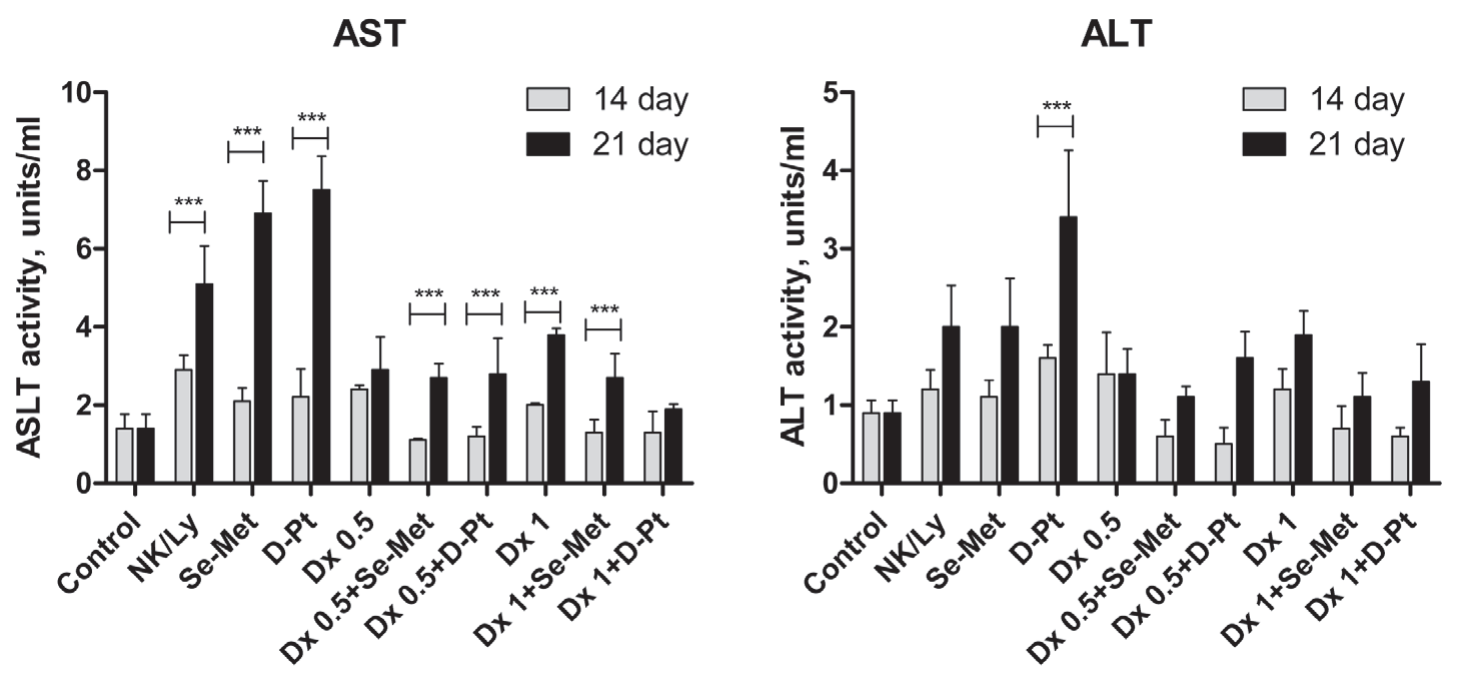
Changes in the activity of aspartate aminotransferase and alanine aminotransferase in NK/Ly lymphoma bearing animals and antioxidant compounds, on the 14th and 21st day after tumor inoculation. ***P < 0.001 related to the 14th day.

At the last stage of our studies, the therapeutic impact of antioxidants on the liver metabolism in Dx-treated animals was analyzed. For this, alanine aminotransferase (ALT) and aspartate aminotransferase (AST) levels were measured, which are considered to be the most widely used hepatotoxicity tests (34). The growth of NK/Ly lymphoma sharply increased AST levels, while action of SeMet and D-Pt alone enhanced it even further on the 21st day, which may indicate potential hepatotoxicity of these dietary supplements only in tumor-bearing animals (Table 2 and Figure 6). Dx possesses moderate hepatotoxicity (35), which developed only on the 21st day after tumor inoculation (Figure 6). However, combined treatment with Dx and studied compounds revealed the opposite tendency – decrease in hepatotoxic effects, caused by Dx, SeMet, and D-Pt (Figure 6). The strongest effect of the tested dietary supplements was observed when they were applied with a high dose of Dx (10 mg/kg), thus confirming their strong tissue-protecting properties. The same tendencies of antioxidants action were observed for alanine aminotransferase (ALT), though they were not statistically significant (Table 2, Figure 6). Analysis of ALT/AST coefficient (De Ritis ratio), which is considered to be a well-known hepatotoxicity index (36), confirmed strong cytoprotective properties for D-Pt, which reversed Dx-induced hepatotoxicity to the control level (Table 1, Figure 6).

**Table 2:** Impact of doxorubicin, selenomethionine, and D-pantethine on ALT/AST levels and De Ritis ratio in the serum of NK/Ly lymphoma bearing mice on 14th day and 21st day after tumor inoculation (M±SD)

Groups	14th day			21st day		
	ALT, units/ml	AST, units/ml	De Ritis ratio	ALT, units/ml	AST, units/ml	De Ritis ratio
Control (healthy)	0.9 ± 0.16	1.4 ± 0.36	**1.5 ± 0.20**	0.9 ± 0.16	1.4 ± 0.36	**1.5 ± 0.20**
Group I (C, NK/Ly)	1.2 ± 0.25	2.9 ± 0.38	**2.4 ± 0.33**	2.00 ± 0.53	5.1 ± 0.97	2.5 ± 0.29*
Group II (Se-Met)	1.1 ± 0.22	2.1 ± 0.34	**1.9 ± 0.09**	2.00 ± 0.62	6.9 ± 0.84	3.4 ± 0.68***
Group III (D-Pt)	1.6 ± 0.17	2.2 ± 0.72	**1.4 ± 0.32**	3.4 ± 0.86	7.5 ± 0.87	**2.2 ± 0.33**
Group IV (Dx 5)	1.4 ± 0.53	2.4 ± 0.11	**1.7 ± 0.61**	1.4 ± 0.32	2.9 ± 0.84	**1.9 ± 0.91**
Group V (Dx 5+Se-Met)	0.6 ± 0.21	1.1 ± 0.05	**1.8 ± 0.79**	1.1 ± 0.14	2.7 ± 0.36	**2.5 ± 0.70**
Group VI (Dx 5+D-Pt)	0.5 ± 0.21	1.2 ± 0.24	**2.4 ± 0.16**	1.6 ± 0.34	2.8 ± 0.91	**1.7 ± 0.14**
Group VII (Dx 10)	1.2 ± 0.26	2.0 ± 0.05	**1.6 ± 0.34**	1.9 ± 0.30	3.8 ± 0.16	**2.0 ± 0.41**
Group VIII (Dx 10+Se-Met)	0.7 ± 0.29	1.3 ± 0.33	**1.9 ± 0.40**	1.1 ± 0.31	2.7 ± 0.61	**2.4 ± 0.50**
Group IX (Dx 10+D-Pt)	0.6 ± 0.11	1.3 ± 0.54	**2.1 ± 0.70**	1.3 ± 0.48	1.9 ± 0.13	**1.5 ± 0.19**

*P < 0.05 related to control, ***P < 0.001 related to control.

A sharp increase in the level of reduced glutathione was observed in the liver of NK/Ly-lymphoma bearing mice already on the 14th day after tumor inoculation (Table 3). This event was also accompanied by a significant increase (*P* < 0.05) in the degree of reduction of its redox potential compared to healthy animals. Doxorubicin treatment had no significant impact on elevated concentration of reduced glutathione in the liver, but joint application of Dx with SeMet or D-Pt led to normalization of these indices to the level of the control animals (Table 3).

**Table 3:** Glutathione level, its redox ratio and redox-potential in the liver of NK/Ly lymphoma bearing mice (M±SD)

Groups	GSH, nmol/mg protein	GSSG, nmol/mg protein	GSH^+^2GSSG, nmol/mg protein	GSH/GSSG	Eh. mV
Control (healthy)	3.94 ± 1.37	0.228 ± 0.091	4.40 ± 1.51	18.6 ± 5.8	-264.1 ± 5.4
Group I (C, NK/Ly)	8.77 ± 0.39*	0.309 ± 0.033	9.39 ± 0.34*	28.7 ± 2.2	-280.9 ± 0.6^●^
Group II (Dx 5)	7.80 ± 0.26*	0.258 ± 0.027	8.32 ± 0.13*	30.6 ± 3.9	-280.2 ± 2.0^●^
Group III (Dx 5+Se-Met)	5.92 ± 0.32^●#^	0.263 ± 0.004	6.45 ± 0.32^●#^	22.6 ± 1.0*	-274.8 ± 1.3*^#^
Group IV (Dx 5+D-Pt)	5.34 ± 0.10^●#^	0.232 ± 0.018^●^	5.80 ± 0.11^●#^	23.9 ± 2.3	-271.8 ± 0.8*^#^

*P < 0.05 related to control; **^●^***P* < 0.05 related to control (NK/Ly); ^#^*P* < 0.05 related to Dx.

Study of activity of two crucial enzymes involved in glutathione biosynthesis – glutathione reductase (GR) and glutathione transferase (GT) – revealed no differences in GR level, and a moderate increase (*P* < 0.05) of GT activity under Dx treatment (Table 4). SeMet and D-Pt in combination with Dx increased GR activity on the 14th day after tumor inoculation, but had no effect on GR level (Table 4). The most prominent changes in activity were observed for selenium-dependent glutathione peroxidase (GPx), the activity of which dropped under Dx treatment (*P* < 0.05 compared to health animals), and then was restored to the control level by SeMet administration. Measurement of overall GPx activity using another assay (30) showed a similar pattern, except the decrease in total GPx activity in untreated tumor-bearing animals, which was not observed in the former assay (Table 4). There was no impact revealed at the action of D-Pt on activity of GPx, which suggests little impact of vitamin B_5_ precursors on glutathione system. Selenium’s role as a crucial component in coenzyme of GPx (15) might explain the observed impact of SeMet on increase on activity of this enzyme, as it may recruit and activate extra number of holoenzymes, already present in cell cytosol.

**Table 4:** Activity of glutathione reductase, glutathione transferase, selenium-dependent glutathione peroxidase and total GPx in the liver of NK/Ly lymphoma bearing animals (M±SD)

Groups	GR, nmol/min/mg protein	GT, nmol/min/mg protein	GPx(Н_2_О_2_), µM GSH/min/mg protein	GPx(t-BOOH), µM GSH/min/mg protein
Control (healthy)	21.3 ± 3.3	507.6 ± 48.8	340.52 ± 34.95	430.71 ± 49.86
Group I (C, NK/Ly)	21.2 ± 2.9	453.7 ± 16.9	389.92 ± 42.57	270.89 ± 74.50^▲^
Group II (Dx 5)	21.6 ± 0.2	584.3 ± 2.7*	261.57 ± 28.54	269.22 ± 29.75^▲^
Group III (Dx 5+Se-Met)	25.4 ± 1.4^#^	593.4 ± 30.3*	370.87 ± 18.41^#^	448.71 ± 83.10
Group IV (Dx 5+D-Pt)	28.8 ± 3.8	592.0 ± 65.4	284.61 ± 49.79	306.07 ± 8.43

^▲^ *P* < 0.05 related to control; * *P* < 0.05 related to control (NK/Ly); ^#^ *P* < 0.05 related to Dx.

Study of coenzyme A system revealed a significant drop in CoA fractions in the liver of tumor-bearing animals, which was not restored by Dx treatment or by combined therapy of Dx and SeMet (Table 5). Only D-Pt (physiological precursor of CoA) restored concentrations of both acid-soluble and free CoA to the control levels, indicating its liver-protective potential, also confirmed by ALT/AST tests (Table 2).

**Table 5:** Impact of selenomethionine, D-pantethine and doxorubicin (Dx) on content of coenzyme A (CoA) fractions (nM/g of tissue) in the liver of NK/Ly lymphoma bearing animals

Groups	Acid-soluble CoA	Short-tailed CoA acyls	Free CoA
Control (healthy)	293 ± 13	141 ± 8	152 ± 7
Group I (C, NK/Ly)	238 ± 14*	119 ± 11	119 ± 6*
Group II (Dx 5)	244 ± 11*	123 ± 7	121 ± 5*
Group III (Dx 5+Se-Met)	247 ± 12*	124 ± 9	122 ± 8*
Group IV (Dx 5+D-Pt)	282 ± 15	136 ± 8	146 ± 7^#^

* *P* < 0.05 related to control; ^#^ *P* < 0.05 related to Dx.

## Discussion

Our study revealed that D-Pt was the most effective dietary supplement, both enhancing the quality of life of NK/Ly lymphoma bearing animals and increasing Dx therapeutic activity *in vivo*. In particular, combination therapy with D-Pt and Dx 5 mg/kg led to tumor remission (survival >60 days) in 100% animals, while in case of Dx 5 mg/kg alone animal survival time was 46-48 days. D-Pt decreased erythropenia and leukocytepenia induced by Dx, lowered neutrophilia and monocytosis, and restored the number of small lymphocytes in blood of tumor-bearing mice almost to the control level (in healthy animals) both on the 14th and 21st day after tumor inoculation. Thus, one can see that D-Pt may possess immunomodulating effect, which is in agreement with other studies (37). Additionally, this antioxidant also had a moderate hepatoprotective effect, revealed in decrease of ALT/AST coefficient and recovery of CoA level in liver of NK/Ly lymphoma bearing animals under Dx treatment. However, NK/Ly lymphoma growth was accompanied by strong erythrocytosis and especially leukocytosis (Figure 4), which significantly hinders interpretation of immunomodulatory activity of the studied antioxidants.

The use of D-Pt (500 mg/kg) together with higher doses of Dx (10 mg/kg) showed less notable results. This antioxidant acted in the same way, decreasing leukocytopenia, erythropenia, monocytosis, and hepatotoxicity caused by Dox, and tumor-derived neutrophilia and leucopenia on the 14th day after tumor inoculation. However, no statistically significant differences in these indices (except leukocytopenia, erythropenia, and monocytosis) were observed on the 21st day after tumor inoculation, meaning that high dose of Dx effectively treated animals with NK/Ly lymphoma without any need for antioxidants. Surprisingly, co-treatment of animals with Dx 10 mg/kg and D-Pt (500 mg/kg) led to remission only in 33% of tumor-bearing mice (survival >60 days), while in *Dx* 10 mg/kg group we observed 100% remission. This phenomenon cannot be explained by tumor regrowth or by cumulative toxicity of Dx, applied together with antioxidants, and thus needs to be clarified in more detail in further studies.

The same tendency, but to lower extent, was observed for SeMet. 600 µg/kg dose of this antioxidant partially reversed all side effects caused by low dose of Dx (5 mg/kg), but the overall survival rate in SeMet+Dx 5 mg/kg group was only 33% compared to 100% in D-Pt+Dx 5 mg/kg group, indicating lower tissue-protective capabilities of selenium compared to vitamin B5 precursors. Combination of SeMet with higher doses of Dx (10 mg/kg) led to the same situation as in the case with D-Pt – remission only in 33% of tumor-bearing mice in contrast to 100% in Dx, and no differences in physiological indices in comparison with Dx alone.

Studies of glutathione, coenzyme A levels, and activity of glutathione-converting enzymes indicate that SeMet and D-Pt realize their tissue-protecting activity in indirect way by influencing glutathione system in the cells. Moreover, flow cytometric studies have not revealed any ROS scavenging effect of SeMet and D-Pt under Dx treatment of Jurkat T-leukemia cells measured by DCFDA/DHE assays (data not presented). This principally distinguishes action of SeMet and D-Pt from other antioxidants already used in clinical medicine (eg, dexrazoxane), which act as iron chelators and directly scavenge toxic superoxide anions produced by Dx (38). Nevertheless, SeMet and D-Pt also have significant benefits compared to dexrazoxane, as they may be administered orally as simple dietary supplements and possess strong immunomodulating properties, in contrast to dexrazoxane, which induces severe leukopenia in cancer patients (38). However, more studies should be performed to understand if the observed therapeutic efficiency of SeMet and D-Pt will be also preserved on other myeloid and solid tumor models. Additionally, tumor own negative impact on health status of experimental animals, therapeutic effect of anticancer drugs toward tumor growth, their potential side effects and finally the potential beneficial effect of antioxidants toward normal cells and tissues should be distinguished and analyzed in tight connection between each other.

In conclusion, selenomethionine and D-pantethine have demonstrated their ability to effectively protect the hematopoietic system and liver of tumor-bearing animals with NK/Ly lymphoma from the side effects of anticancer drug Dx, which was revealed by normalization of blood indexes, animal weight, and ALT/AST activity. In overall, D-Pt demonstrated better tissue-protective and even therapeutic activity compared to SeMet, leading to a significant increase in survival of NK/Ly lymphoma bearing animals under Dx treatment. However, tissue-protecting properties of the studied antioxidants may also depend on the dosage of anticancer drug. Both compounds enhance therapeutic action of low doses of Dx, but fail to increase survival of animals treated with high dose of Dx, though in both cases SeMet and D-Pt increase their quality of life. More studies on pharmacokinetics and potential interaction of antioxidants with doxorubicin should be carried out before making any conclusions on their potential application in clinical practice. This will allow us to better understand if SeMet and D-Pt can enhance the therapeutic effect of Dx on myeloid tumors in cancer patients and thus increase their median survival time, which may be of high importance for development of novel schemes of cancer therapy based on the combined use of anticancer drugs and antioxidant food supplements.
